# Early-Life Supplementation Enhances Gastrointestinal Immunity and Microbiota in Young Rats

**DOI:** 10.3390/foods13132058

**Published:** 2024-06-28

**Authors:** Laura Sáez-Fuertes, Garyfallia Kapravelou, Blanca Grases-Pintó, Manuel Bernabeu, Karen Knipping, Johan Garssen, Raphaëlle Bourdet-Sicard, Margarida Castell, María José Rodríguez-Lagunas, María Carmen Collado, Francisco José Pérez-Cano

**Affiliations:** 1 Physiology Section, Department of Biochemistry and Physiology, Faculty of Pharmacy and Food Science, University of Barcelona (UB), 08028 Barcelona, Spain; laurasaezfuertes@ub.edu (L.S.-F.); gkapravelou@ub.edu (G.K.); blancagrases@ub.edu (B.G.-P.); margaridacastell@ub.edu (M.C.); franciscoperez@ub.edu (F.J.P.-C.); 2 Nutrition and Food Safety Research Institute (INSA-UB), 08921 Santa Coloma de Gramenet, Spain; 3 Institute of Agrochemisty and Food Technology-National Research Council (IATA-CSIC), 46980 Valencia, Spain; mbernabeu@iata.csic.es (M.B.); mcolam@iata.csic.es (M.C.C.); 4 Danone Research & Innovation, 3584 Utrecht, The Netherlands; karen.knipping@ausnutria.nl (K.K.); johan.garssen@danone.com (J.G.); 5 Division of Pharmacology, Faculty of Science, Utrecht Institute for Pharmaceutical Sciences, 3584 CG Utrecht, The Netherlands; 6 Danone Global Research & Innovation Center, Gif, 91190 Gif-sur-Yvette, France; raphaelle.bourdet-sicard@danone.com; 7 Center for Biomedical Research Network for the Physiopathology of Obesity and Nutrition (CIBEROBN), Instituto de Salud Carlos III, 28029 Madrid, Spain

**Keywords:** *Bifidobacterium breve* M-16V, galacto-oligosaccharides (GOS), fructo-oligosaccharides (FOS), immunonutrition, immune system

## Abstract

Immunonutrition, which focuses on specific nutrients in breast milk and post-weaning diets, plays a crucial role in supporting infants’ immune system development. This study explored the impact of maternal supplementation with *Bifidobacterium breve* M-16V and a combination of short-chain galacto-oligosaccharide (scGOS) and long-chain fructo-oligosaccharide (lcFOS) from pregnancy through lactation, extending into the early childhood of the offspring. The synbiotic supplementation’s effects were examined at both mucosal and systemic levels. While the supplementation did not influence their overall growth, water intake, or food consumption, a trophic effect was observed in the small intestine, enhancing its weight, length, width, and microscopic structures. A gene expression analysis indicated a reduction in *FcRn* and *Blimp1* and an increase in *Zo1* and *Tlr9*, suggesting enhanced maturation and barrier function. Intestinal immunoglobulin (Ig) A levels remained unaffected, while cecal IgA levels decreased. The synbiotic supplementation led to an increased abundance of total bacteria and Ig-coated bacteria in the cecum. The abundance of *Bifidobacterium* increased in both the intestine and cecum. Short-chain fatty acid production decreased in the intestine but increased in the cecum due to the synbiotic supplementation. Systemically, the Ig profiles remained unaffected. In conclusion, maternal synbiotic supplementation during gestation, lactation, and early life is established as a new strategy to improve the maturation and functionality of the gastrointestinal barrier. Additionally, it participates in the microbiota colonization of the gut, leading to a healthier composition.

## 1. Introduction

Immunonutrition is defined as the capacity of specific nutrients to regulate or influence the activity of the immune system [1]. Immunonutrients include vitamins, minerals, amino acids, and fatty acids that play a direct role in supporting or regulating the immune system [2]. Although some of these components in the maternal diet during gestation reach the fetus through the placenta, breast milk (BM) serves as the primary source of immunonutrients for neonates and infants. The bioactive compounds of the BM stimulate the maturation of the immune system, contribute to immune tolerance, and aid in the colonization of the intestinal microbiome [3,4]. Human BM is considered the optimal nutrition for infants during the first 6 months of life and facilitates immune system development. After weaning, healthy nutritional habits continue to enhance the infant’s immune system, reducing his or her susceptibility to infections and diseases [5]. According to this, immunonutrition aims to enhance the development of the infant before birth and during early childhood. This corresponds to the first 1000 days of life, encompassing the infant’s life from conception until 2 years of age. During this critical time, optimal growth and cognitive development occur, which are essential for long-term health. A crucial aspect of this development is the establishment of a healthy gut microbiota, which plays a vital role in nutrient absorption, immune function, and protection against pathogens.

Probiotics, prebiotics, and synbiotics are not classified as immunonutrients, but they exert a modulatory effect on the immune system [6,7]. The immunomodulatory effects of pro-, pre-, and synbiotics are strain-specific and dose-dependent [6]. *Bifidobacteria* and *lactobacilli* are commonly used probiotic strains that help reduce gut inflammation and maintain intestinal balance [8,9]. Prebiotics, among other actions, serve as specific substrates for the intestinal microbiota, encouraging its proliferation and avoiding pathogen colonization through their antiadhesive properties. Short-chain galacto-oligosaccharides (scGOSs) and long-chain fructo-oligosaccharides (lcFOSs) are common prebiotics known for promoting the growth of beneficial gut bacteria when added to infant formulas [9,10]. Synbiotics, a blend of pro- and prebiotics, yield a synergistic effect by ensuring the viability of the probiotic bacteria and their growth stimulation by the delivered substrates. Supplementation with synbiotics has been linked to a reduction in the incidence of eczema, asthma development, infectious diseases, respiratory issues, and dysentery [11,12,13,14,15]. These findings emphasize the essential role of these compounds in strengthening and optimizing the immune system, consequently promoting overall health and well-being.

Supplementing maternal diets with synbiotics during both gestation and lactation positively impacts maternal immune health and contributes to the development of the neonatal immune system [16,17]. Specifically, synbiotics modulate the bioactive composition of the BM, contributing to the passive immunization of the infant [18]. In our research, we assessed the impact of maternal synbiotic supplementation from gestation through lactation and continuing into the offspring’s early childhood for one week. The synbiotic mix consisted of *Bifidobacterium breve* M-16V and scGOS/lcFOS. The overall period we studied in rats is the equivalent of the first 1000 days of life in humans, considered as essential in infant development [19,20]. We examined the supplementation’s immunomodulatory effects on both systemic and mucosal immunity, and its influence on various lymphocyte populations as well as on the microbiota composition, diversity, and activity.

## 2. Materials and Methods

### 2.1. Animals and Experimental Design

Sixteen female and eight male Lewis rats were obtained from Janvier Labs in La Plaine Saint Denis Cedex, France. After one week of acclimatization, the females were placed in the males’ cages for one week for mating. Then the females were separated and placed in individual cages.

Afterwards, the female rats were divided into two experimental groups (*n* = 8/group): reference (REF) and synbiotic (SYN). Animals from the two groups were supplemented daily with a synbiotic during gestation (21 days) and lactation (21 days). Pups were allowed to be born naturally. After weaning, the pups were directly supplemented from day 21 to day 28 of life with the same mix. The day of birth was designated as day 1 for the pups and litters were unified up to 10 pups. From these, 3 pups/litter were kept until day 28 of life, which is described in the present study (Supplementary Figure S1). The pups had free access to their mother’s nipples for nursing (until day 21) and were also provided with the rat diet used in the study. Throughout the study, the rats had unrestricted access to a commercial diet formulated according to the American Institute of Nutrition 93G formulation [21]. They were also provided with water *ad libitum*. 

The synbiotic was administered daily by oral gavage to the dams during gestation and lactation and to the pups after weaning. The synbiotic was freshly prepared by mixing *Bifidobacterium breve* M-16V (10^9^ CFU) with scGOS/lcFOS in a physiological saline solution. For the dams, the dose of scGOS/lcFOS was approximately 2% of the established daily food intake of 40 g. The administered volume was fixed at 1 mL during gestation and at 1.5 mL during lactation. For the pups, from day 21 to day 28 of life, the dose was fixed at 2 × 10^7^ UFC/rat/day, administered in a volume of 0.2 mL. The REF dams and the REF pups received an equivalent volume of a saline solution under the same conditions. All supplements were kindly provided by Danone Research & Innovation (Utrecht, The Netherlands). 

The animals’ room conditions (temperature and humidity) were controlled. The room followed a 12 h light and 12 h dark cycle within a negative pressure chamber at the Diagonal Campus Animal Facility of the Faculty of Pharmacy and Food Science at the University of Barcelona (UB). The experimental procedures conducted in this study were carried out with the necessary ethical approvals. This study received approval from the Ethics Committee for Animal Experimentation (CEEA) of the University of Barcelona (Ref. 240/19) and from the Catalan Government (Ref. 10933). 

### 2.2. Sampling and Processing

From birth until day 28 of the pups’ life, animal weight was measured, and food intake was followed from weaning (day 21) to day 28. Then the pups were anesthetized with ketamine (90 mg/kg) and xylazine (10 mg/kg; Bayer A.G., Leverkusen, Germany). They were then measured to evaluate their overall development according to the Body Mass Index (BMI) ((weight/length^2^ (g/cm^2^)) and the Lee index (weight^0.33^/length) × 1000 (g^0.33^/cm)). 

Cardiac exsanguination was performed to obtain blood samples. Intestinal samples, adipose tissues (epididymal, parametric, dorsal, retroperitoneal, and inguinal for white adipose tissue (WAT) and brown adipose tissue (BAT)), cecal content (CC), salivary gland (SG), mesenteric lymph nodes (MLNs), and spleen were collected and immediately processed or stored at −20 °C or −80 °C for future analyses. 

Blood samples were analyzed using an automated hematologic analyzer (Spincell, MonLab Laboratories, Barcelona, Spain) and centrifuged to obtain plasma.

Small intestine (SI) and adipose tissue were collected for histological evaluation. Additionally, the central section of the SI was collected for gene expression analysis. One centimeter of the central section of the SI was embedded in RNA later (Ambion, Life Technology, Madrid, Spain), kept at 4 °C for 24 h, and then stored at −20 °C. The remaining portion of the proximal SI was opened lengthwise and incubated by shaking (37 °C, 10 min) with phosphate-buffered saline (PBS), and the supernatant (gut wash (GW)) was recovered for immunoglobulin (Ig) quantification. 

The content of the distal part of the SI (intestinal content, IC) and the CC were collected for microbiota analysis to characterize their different population proportions and their metabolite production

Part of the MLNs and the spleen were processed as previously described to obtain splenic and MLN lymphocytes [22]. Briefly, tissues were ground using a 40 µm mesh cell strainer (Thermo Fisher Scientific, Barcelona, Spain) by using enriched Roswell Park Memorial Institute (RPMI) 1640 medium (Sigma-Aldrich, Madrid, Spain). The obtained suspension was centrifuged, and the resulting pellet was resuspended in enriched RPMI. 

The remaining MLNs, SG, and CC were frozen. Then they were thawed and homogenized for Ig quantification using enzyme-linked immunosorbent assay (ELISA), ProcartaPlex™ Multiplex immunoassay (eBioscience, San Diego, CA, USA), or flow cytometry. The weight of different organs was recorded, including thymus, spleen, liver, heart, kidney, large intestine, and SI (length and width were also measured).

### 2.3. Tissue Histology

The chosen sections of the SI and both BAT and WAT (epidydimal or parametric sections for males or females, respectively, were chosen as being representative of WAT) were initially fixed in a 4% buffered formaldehyde solution for 24 h at room temperature. Subsequently, the samples were rinsed in PBS solution and dehydrated using a series of graded ethanol solutions (70%, 90%, and 100%) and permeated in xylene (Panreac Química SLU, Barcelona, Spain). Following this, the samples were embedded in melted paraffin (Merck, Madrid, Spain). Paraffin sections that were 5 µm thick were obtained with a microtome (Leica biosystems, Nußloch, Germany) and stained using hematoxylin–eosin (HE). Samples were examined using an Olympus BX41 microscope and Olympus XC50 camera. Representative photos were taken at 20× magnification for WAT, 40× for BAT, and 10× for intestine sections. All histological samples were analyzed using Image J 1.53p software (National Institute of Mental Health in Bethesda, MD, USA).

### 2.4. Gene Expression Analysis

SI samples stored in RNAlater were thawed for RNA extraction and gene expression analysis. Samples were homogenized using lysing matrix tubes and a FastPrep-24 instrument (MP Biomedicals, Illkirch, France), as previously described [23]. RNA extraction was performed following the manufacturer’s protocol using the RNeasy Mini Kit (Qiagen, Madrid, Spain) and quantified with a NanoPhotometer (BioNova Scientific S.L., Fremont, CA, USA). cDNA was obtained using TaqMan Reverse Transcription Reagents (Applied Biosystems, AB, Weiterstadt, Germany). Then, real-time (RT)-PCR was performed with an ABI Prism 7900HT quantitative RT-PCR system (AB) in the Scientific and Technological Centers of the University of Barcelona (CCiT-UB). 

The specific TaqMan primers (AB) used can be found in Supplementary Table S1. The housekeeping gene *Gusb* (β-glucuronidase) was used to normalize the obtained results. Data were analyzed using the −2ΔΔCt method, as previously described [24]. The data are presented as a percentage of expression in each experimental group, normalized to the mean value obtained for the REF group, which was set at 100%.

The detection of the *B. breve* M 16-V was performed for the CC after the DNA extraction, as previously described [25]. The TaqMan-based forward, reverse, and probe assays were previously designed by Phavichitr et al. [26].

### 2.5. Immunoglobulin Quantification

Plasma, GW, and homogenized MLNs, SG, and CC were used for Ig quantification. Secretory (s)IgA was quantified by ELISA. Additionally, IgM was also evaluated in GW. sIgA and IgM were quantified following the previous procedures [27]. Absorbance data were acquired using a microplate photometer (Labsystems Multiskan, Helsinki, Finland) at 495 nm. Results were analyzed using Multiskan Ascent v2.6 software (Thermo Fisher Scientific SLU, Barcelona, Spain). The lower limits of detection were 1.95 ng/mL for sIgA and IgM.

In plasma and homogenized MLNs and SG, a ProcartaPlex™ Multiplex immunoassay was performed to quantify IgA, IgM, IgG, and IgG isotypes (IgG1, IgG2a, IgG2b, and IgG2c). Briefly, 96-well flat-bottom plates were used to prepare samples following the manufacturer’s instructions, as described in previous studies [28]. Data were acquired using MAGPIX® analyzer (Luminex Corporation, Austin, TX, USA) at the Cytometry Service of the CCiT-UB. The lower limits of detection of each Ig can be found in Supplementary Table S2. Th1 and Th2 responses were evaluated by adding the levels of IgG subtypes: IgG2b + IgG2c and IgG1 + IgG2a, respectively.

### 2.6. Cecal Bacteria and Ig-Coated Bacteria Analysis

The proportion of cecal bacteria and Ig-coated bacteria (Ig-CB) was determined as previously described [29]. Data were acquired using Cytek Aurora (Cytek Biosciences, Inc., Fremont, CA, USA) flow cytometry equipment at the CCiT-UB. FlowJo v.10 software (Tree Star, Inc., Ashland, OR, USA) was used to analyze data by following the protocol described by Massot et al. [30].

### 2.7. Cell Subset Staining and Flow Cytometry Analysis

Mesenteric and splenic lymphocyte subsets were characterized by flow cytometry analysis using fluorescent mouse anti-rat monoclonal antibodies (mAbs) conjugated to different fluorochromes, as previously described [31]. Data were acquired in the CCiT-UB using a Gallios^TM^ Cytometer (Beckman Coulter, Miami, FL, United States) and analyzed using FlowJo v.10 software. 

### 2.8. Short-Chain Fatty Acid Quantification

Short-chain fatty acid (SCFA) analysis was performed using gas chromatography–mass spectrometry (GC-MS), following the method described by Eberhart et al. [32]. An internal standard solution (3-methylvaleric acid) was added to the samples, which were processed and finally centrifuged at 1500× *g* for 2 min at 4 °C, according to the protocol. The final supernatant was collected, filter sterilized (0.22 μm PES size filter, Sarstedt SA, Nümbrecht, Germany), and injected in the Agilent GC 7890B–5977B GC-MS with a multipurpose sampler (Gerstel MPS, Mülheim, Germany). The GC column used was an Agilent DB-FATWAX that was 30 m × 0.25 mm × 0.25 μm, operating in split mode (20:1). The oven temperature program was set as follows: 100 °C for 3 min, ramped up to 100 °C at a rate of 5 °C min^−1^, then to 150 °C for 1 min, further ramped up to 200 °C at a rate of 20 °C min^−1^, and finally held at 200 °C for 5 min. Helium was used as the carrier gas at a flow rate of 1 mL min^−1^, with an inlet temperature of 250 °C. The injection volume was 2 μL. Standard curves for acetate, butyrate, and propionate were used for quantifying the SCFAs.

### 2.9. Microbiota Analysis by 16S rRNA Amplicon Sequencing

Total DNA was isolated from the intestinal content (IC) and CC (100–200 mg) using an automated assisted method based on magnetic beads (Maxwell® RSC Instrument coupled with Maxwell RSC Pure Food GMO and authentication kit, Promega, Madrid, Spain) following the manufacturer’s instructions, with prior treatments conducted to improve the DNA extraction. In brief, samples were treated with lysozyme (20 mg/mL) and mutanolysin (5 U/mL) for 60 min at 37 °C, and preliminary cell disruption was induced with a FastPrep 24-5 G bead beating homogenizer (MP Biomedicals). After DNA purification (Purification Kit, Macherey-Nagel, Duren, Germany), DNA concentration was measured using a Qubit® 2.0 fluorometer (Life Technology, Carlsbad, CA, USA). 

Libraries targeting the amplicon V3–V4 variable region of the 16S rRNA gene were prepared following the 16S rDNA gene Metagenomic Sequencing Library Preparation Illumina protocol (Cod. 15044223 Rev. A) and then sequenced using 2 × 300 bp paired-end run on a MiSeq-Illumina platform (FISABIO sequencing service, Valencia, Spain). Negative and positive mock communities were also included. Raw reads were then processed with the integrated dada2 [33] method for denoising, amplicon sequence variance (ASV) clustering, and chimera removal. The resultant ASVs were then taxonomically assigned using Silva v.138. 

### 2.10. Statistical Analysis

The statistical analysis was performed using SPSS Statistics 22.0 software (SPSS Inc., Chicago, IL, USA). Normality and variance homogeneity were assessed using the Shapiro–Wilk and Levene tests, respectively. When the data exhibited normal and homogeneous distributions, a one-way ANOVA was performed for analysis. In cases in which the data did not adhere to normal and equal distribution assumptions, the Kruskal–Wallis test was used to identify significant differences (*p* < 0.05). To explore variable correlations, the Spearman correlation coefficient was calculated.

To identify clusters of sample similarities based on immune factor composition, non-metric multidimensional scaling (NMDS) was employed in Rstudio, utilizing the ‘vegan’ package [34]. To assess the association of factors with the ordination of samples in the NMDS plot, the ‘envfit’ function was used. Statistical significance was considered when the *p*-value was < 0.05. 

Regarding the microbiota analysis, no rarefaction was performed, samples with less than 4500 reads were removed, and data were normalized using centered log-ratio (CLR). Beta diversity analysis was based on the Bray–Curtis distance matrix, and permutational analysis of variance (PERMANOVA) was performed. Chao1 and Shannon alpha-diversity indexes were also calculated, and differences by group were assessed by Mann–Whitney and/or Kruskal–Wallis non-parametric tests. In addition, the Kruskal–Wallis test on the CLR normalized data was also assessed using the Benjamini–Hochberg false discovery rate (FDR) correction. Negative binomial regression as implemented using the DESeq2 tool was used in differential abundance analysis to estimate the fold-change in taxa (genus) [35].

## 3. Results

### 3.1. Body Weight and Intake Consumption

**Figure 1:**
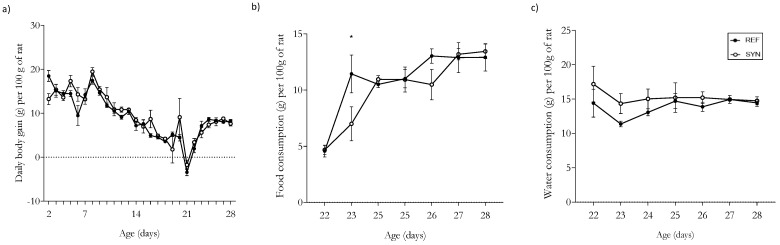
Growth trend until day 28. (**a**) Daily body mass gain; (**b**) Food consumption from day 2 to day 28 of life; (**c**) Water intake from day 2 to day 28 of life. Results are expressed as mean ± standard error of the mean (SEM). Statistical differences: * *p* < 0.05 vs. REF (*n* = 9–11).

The animals’ growth was monitored daily from day 2 until the last day of the study (day 28 of their life). The pups weighed ~5–7 g at birth (day 2), ~33–37 g at the end of the suckling period (day 21), and about 61–65 g the week after weaning (day 28) (Figure 1). From birth, the SYN pups showed a similar growth pattern to that of the REF group. After the weaning period (as measured on day 21), both groups showed a decrease in body weight as a consequence of the separation from their dams (Figure 1a). After the pups were separated from the dams, their water intake and food consumption were monitored daily. Their food consumption increased in line with their growth from ≈5 g at day 21 to ≈12 g at day 28 (Figure 1b), and their water intake was constant throughout the study, with overall values of ≈13 mL (Figure 1c). Generally, the synbiotic supplementation did not modify their total water intake or food consumption. 

### 3.2. Growth Parameters and Organ Weight

On day 28 of their life, the pups were measured and weighed to calculate growth-associated parameters, including their BMI and Lee index values (Table 1). Additionally, the weights of different organs were assessed, and the majority showed no significant changes. However, the synbiotic supplementation triggered a trophic effect in the SI, leading to an increase in its weight, length, width, and area.

### 3.3. Hematologic Variables

After the synbiotic supplementation (day 28), the rats’ hematological variables were assessed (Table 2). The synbiotic supplementation lowered their total leukocyte population counts, primarily attributable to reductions in lymphocytes and granulocytes, without affecting their erythrocyte and platelet measurements.

### 3.4. Adipose Tissue

**Figure 2:**
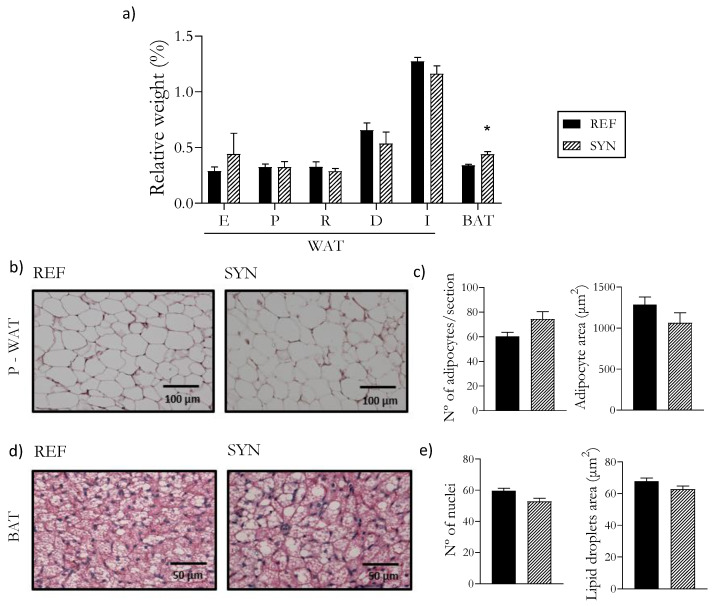
Impact of synbiotic supplementation on the adipose tissue at day 28. (**a**) Relative weights of different sections of adipose tissue; (**b**) Representative histological sections of WAT (epidydimal or parametric sections, for males or females, respectively, were chosen as being representative of the WAT); (**c**) Analysis of WAT: adipocyte area and number of adipocytes; (**d**) Representative histological sections of BAT; (**e**) Analysis of BAT: number of nuclei and area of lipid droplets (LDs). Histological sections (**b**,**d**) were stained with hematoxylin–eosin. Images were taken at 40× and 20× magnification, respectively. Data (**a**,**c**,**e**) are expressed as mean ± SEM. Statistical differences: * *p* < 0.05 vs. REF (*n* = 9–11). WAT, white adipose tissue; BAT, brown adipose tissue; E, epididymal; P, parametric; R, retroperitoneal; D, dorsal; I, Inguinal.

The influence of the administration of the synbiotic on the rats’ adipose tissue examined on day 28 of their life (Figure 2) revealed an increase in the relative weight of BAT (Figure 2a). A histological analysis using HE staining on both their WAT (Figure 2b,c) and BAT (Figure 2d,e) revealed no observable alterations associated with the synbiotic supplementation.

### 3.5. Intestinal Morphology and Maturation

**Figure 3:**
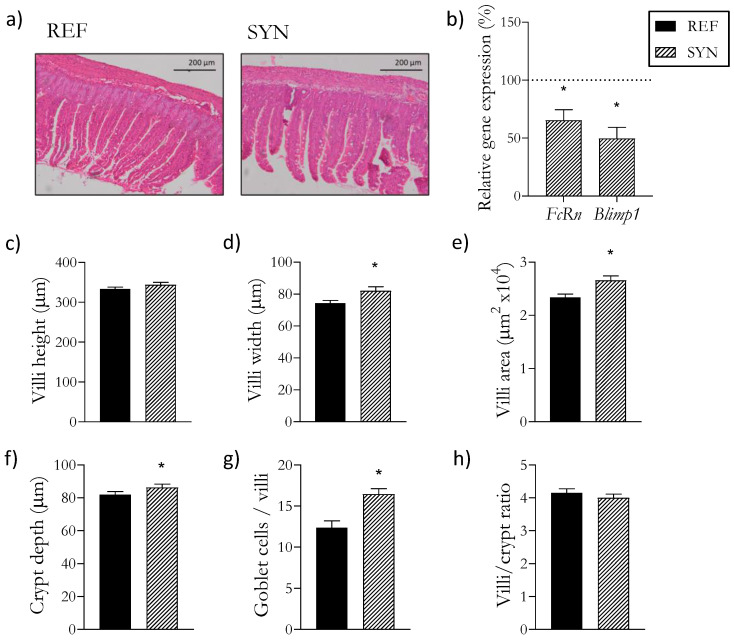
Influence of SYN supplementation on the pups’ intestine at day 28. (**a**) Representative sections of the small intestine stained with hematoxylin and eosin, 10×; (**b**) Relative gene expression analysis in the small intestine of immunological proteins; (**c**) Villi height; (**d**) Villi width; (**e**) Villi area; (**f**) Crypt depth; (**g**) Ratio of villi/crypt; (**h**) Ratio of goblet cells/villi. Relative gene expression (**b**) was calculated with respect to REF, which corresponded to 100% of transcription (represented with a horizontal dotted line). Results (**c**–**h**) are expressed as mean ± SEM. Statistical differences: * *p* < 0.05 vs. REF (*n* = 9–11). *Blimp1*, B lymphocyte-induced maturation protein-1; *FcRn*, neonatal Fc receptor.

Intestinal maturation was examined at both biochemical and microscopic levels (Figure 3). *FcRn* and *Blimp1*, recognized as reliable indicators of intestinal maturation [36], displayed reduced gene expression after the synbiotic supplementation, thus indicating a higher level of maturation. In terms of morphological changes resulting from the supplementation, the SYN pups exhibited an increase in villi width and area, a greater crypt depth, and a higher number of goblet cells per villi. 

### 3.6. Intestinal Expression of Barrier and Crosstalk Genes

**Figure 4:**
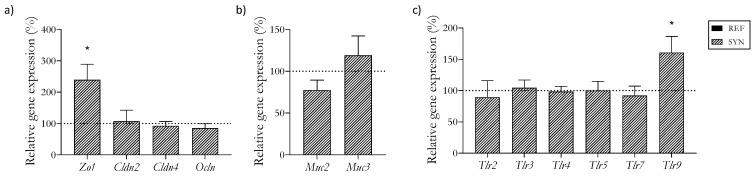
Impact of SYN supplementation on the small intestine gene expression at day 28. (**a**) Relative gene expression of (**a**) tight junction proteins; (**b**) mucins; and (**c**) toll-like receptors. Relative gene expression was calculated with respect to REF, which corresponded to 100% of transcription (represented with a horizontal dotted line). Statistical differences: * *p* < 0.05 vs. REF. (*n* = 9–11). *Zo1*, zonula occludens-1; *Cldn2*, claudin 2; *Cldn4*, claudin 4; *Ocln*, occludin; *Muc2*, mucin2; *Muc3*, mucin3; *Tlr*, toll-like receptor.

The supplementation increased the tight junction (TJ) protein *Zo-1*‘s mRNA levels, whereas it did not modify *Cldn2*, *Clnd4*, or *Ocln* (Figure 4a). The genes associated with mucus production, specifically *Muc2* and *Muc3*, remained unaffected (Figure 4b). Among the multiple evaluated TLRs, only *Tlr9* exhibited a gene expression increase after the supplementation (Figure 4c). 

### 3.7. Gastrointestinal Ig Profile 

**Figure 5:**
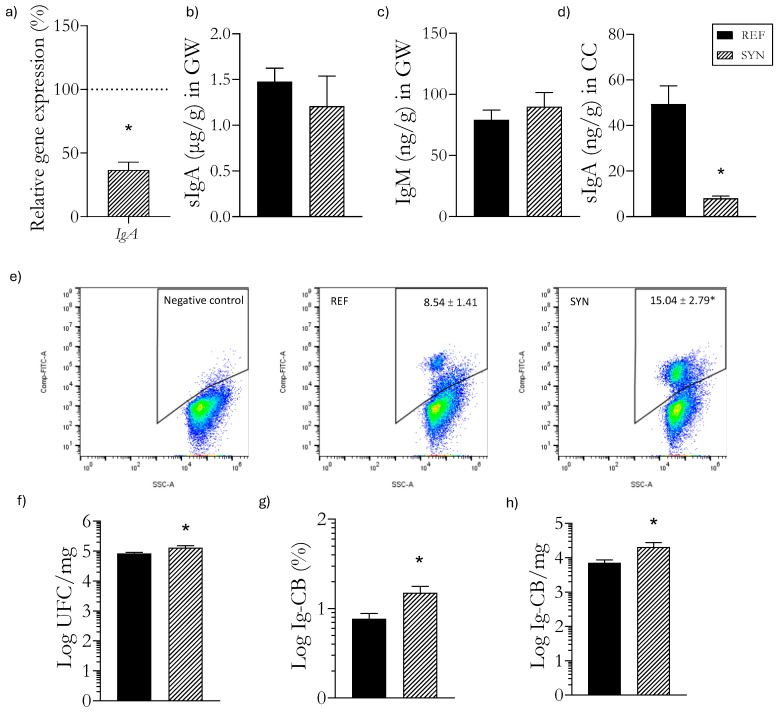
Impact of synbiotic supplementation at day 28 of life on the gastrointestinal tract. (**a**) IgA expression levels in the GW; (**b**) Protein sIgA levels in the GW; (**c**) Protein IgM levels in the GW; (**d**) Protein sIgA levels in the cecum; (**e**) Representative dot-plots of the proportion of Ig-CB in the cecum; (**f**) Counts of total bacteria of the cecum; (**g**) Proportion of Ig-CB of the cecum; (**h**) Total counts of Ig-CB in the cecum. Relative gene expression (**a**) was calculated with respect to REF, which corresponded to 100% of transcription (represented with a horizontal dotted line). Data (**b**–**h**) are expressed as mean ± SEM. Statistical differences: * *p* < 0.05 vs. REF (*n* = 9–11). sIgA, secretory immunoglobulin *A*; GW, gut wash; CC, cecal content; Ig-CB, immunoglobulin-coated bacteria.

The effects of the synbiotic supplementation were analyzed regarding the rats’ Ig profile in the gastrointestinal tract (Figure 5). In their GW, sIgA was quantified at the gene and protein levels. The IgA mRNA levels were reduced after the SYN supplementation (Figure 5a). However, the total sIgA protein remained unaffected (Figure 5b). The IgM levels were not modified after the synbiotic supplementation either (Figure 5c). To further understand the impact of the supplementation on sIgA functionality, an analysis of the sIgA content was conducted in the cecum. A reduction in the total levels of sIgA after the supplementation was observed (Figure 5d). Thus, cecal sIgA plays a crucial role in binding to cecal bacteria to neutralize pathogenic bacteria and maintain intestinal homeostasis [37,38]. The proportion of IgA-CB was also evaluated, and our results indicated that the maternal and early-life infant synbiotic intervention led to an increase in the total cecal bacteria and in the Ig-CB proportion, suggesting that the lower levels of free Ig are due to the higher presence of coating bacteria (Figure 5f–h). 

### 3.8. Microbiota

**Figure 6:**
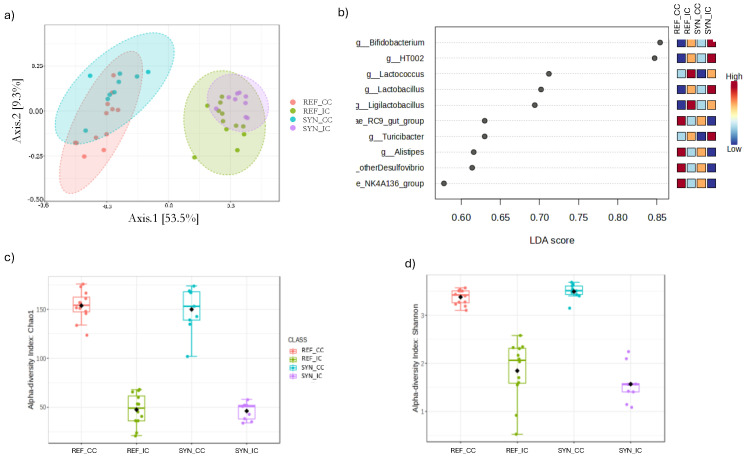
(**a**) Beta-diversity in IC and CC microbiota depending on SYN intervention at day 28; (**b**) Linear discriminant analysis (LDA) effect size (LEfSe) plot of taxonomic genera identified for CC and IC; Alpha-diversity indexes (**c**) (Shannon index) and (**d**) richness (Chao1 index) for CC and IC. Statistical testing was performed by PERMANOVA using Bray–Curtis distances, and the Mann–Whitney test was used for alpha-diversity indexes (*n* = 9–11). Intestinal content, IC; cecal content, CC.

**Figure 7:**
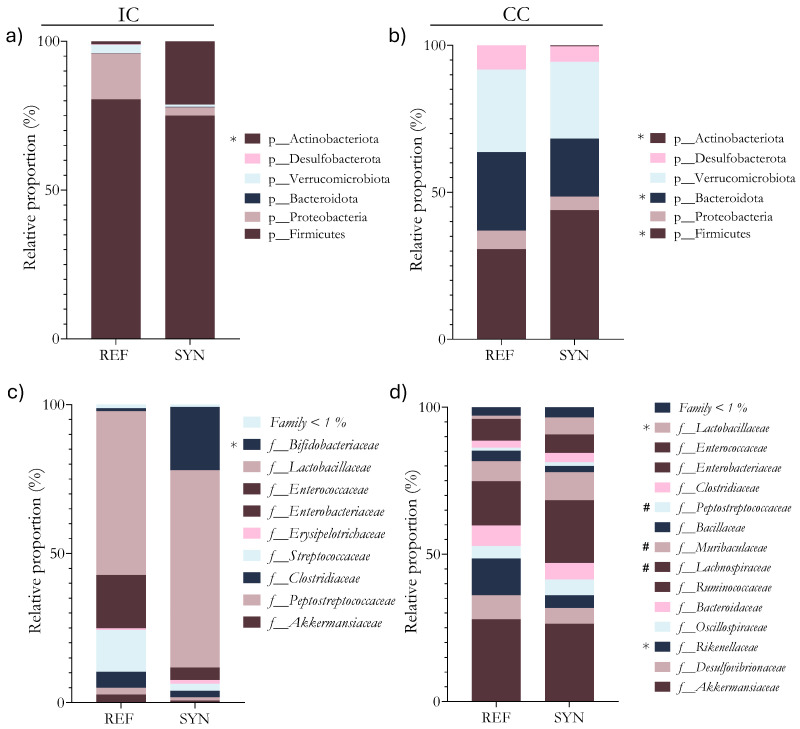

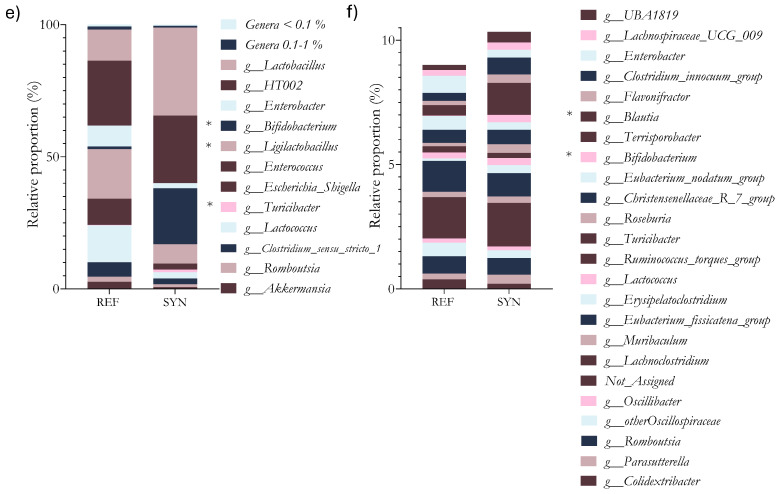
Microbiota composition in terms of the intestinal content (IC) and cecal content (CC) at day 28. Relative abundance of phylum in (**a**) intestine and (**b**) cecum; Relative family abundance in (**c**) intestine and (**d**) cecum; Relative genus abundance in (**e**) intestine and (**f**) cecum. Results are expressed as relative proportions of population. Statistical differences: * *p*< 0.05 vs. REF; ^#^ *p*< 0.1 (*n* = 9–11).

Differences in the microbiota composition were observed between the REF and SYN groups, as well as between sample types (IC and CC) (Figure 6 and Figure 7).

The beta-diversity and alpha-diversity analysis showed significant differences depending on the sample type (intestine vs. cecum) and on the intervention (REF vs. SYN). In detail, regarding the beta-diversity, two general distinct microbial clusters depending on the sample type (CC vs. IC) were found, accounting for 62.8% of the total variation (PERMANOVA test F-value: 18.98; R-squared: 0.60; *p*-value: 0.001, Figure 6a). In addition, when the groups were compared considering the intervention, we observed a significant impact of the SYN intervention on the cecum (PERMANOVA test F-value: 4.80; R-squared: 0.201; *p* = 0.001) but not on the microbiota from the small IC (PERMANOVA F-value: 1.68; R-squared: 0.0813; *p* = 0.125) (Supplementary Figure S2a,b). DESEq tests showed that for the CC, an enrichment of *Bifidobacterium* (FDR *p* = 0.002) and *Lactobacillus* sp. oral clone HT002 (FDR *p* < 0.0001) was found in addition to a significant reduction in the genera *Rikenellaceae*_RC9_gut_group (FDR *p* = 0.002), *Prevotellaceae*_UCG_001 (FDR *p* = 0.021), other *Lachnospiraceae* (FDR *p* = 0.007), and *Intestinimonas* (FDR *p* = 0.1541). For the IC, the SYN intervention increased the presence of *Turicibacter* (FDR *p* = 0.005), *Hungatella* (FDR *p* = 0.007), *Lachnoclostridium* (FDR *p* = 0.085), and *Erysipelatoclostridium* (FDR *p* = 0.192) and decreased the presence of *Escherichia_Shigella* (FDR *p* < 0.0001), *Lactococcus* (FDR *p* = 0.019), and *Enterobacter* (FDR *p* = 0.027) (Figure 6b and Supplementary Table S3). The LEfSe analysis also demonstrated the role of these microbial genera depending on the SYN intervention for both the CC and IC (Supplementary Figure S2c).

Regarding alpha-diversity metrics, the SYN intervention did not influence either the microbial richness (as measured by the Chao1 index) or the microbial diversity (as measured by the Shannon index) in the cecum (CC, *p* = 0.943 and *p* = 0.149, respectively) and in the intestine (IC, *p* = 0.654 and *p* = 0.149, respectively) (Figure 6c,d). Moreover, it should be mentioned that higher significant levels of microbial diversity and richness were observed for the CC compared to the IC.

Taking into account the different bacterial proportions, regarding the IC, the proportion of the Actinobacteria phylum (mainly the *Bifidobacterium* genus) was significantly increased after the SYN supplementation (Figure 7a). The *Bifidobacteriaceae* family was significantly increased in the SYN group (Figure 7c). At the genus level, *Turicibacter* and *Bidifobacterium* were increased while *Ligilactobacillus* was reduced in the SYN pups (Figure 7e). 

Regarding the CC, the SYN intervention increased the relative abundance of members from the Firmicutes and Actinobacteria phyla and reduced the relative abundance of members from the Bacteroidota phyla (Figure 7b). At the family levels, a significative reduction in the abundance of *Rikenecellaceae* members and an increase in *Lactobacillaceae* members were observed in the pups in the SYN group (Figure 7d). In the minority families (<1%), a significant increase in *Bifidobacteriaceae* and a reduction in *Staphylococaceae* were also found in the SYN group. Regarding genera proportions, changes in the minority populations (0.1–1%) were found (Figure 7f). After the SYN supplementation, the *Bifidobacterium*, *Blautia*, *Faecalibaculum*, and *Lactobacillus* proportions were significantly increased. In addition, *B. breve* M-16V was detected by PCR testing cecum samples from the SYN group after one week of supplementation with 2–3 × 10^9^ UFC/mg in 100% of the animals. These results are in line with the increase in the *Bifidobacterium* proportions found.

### 3.9. SCFA Profile

**Figure 8:**
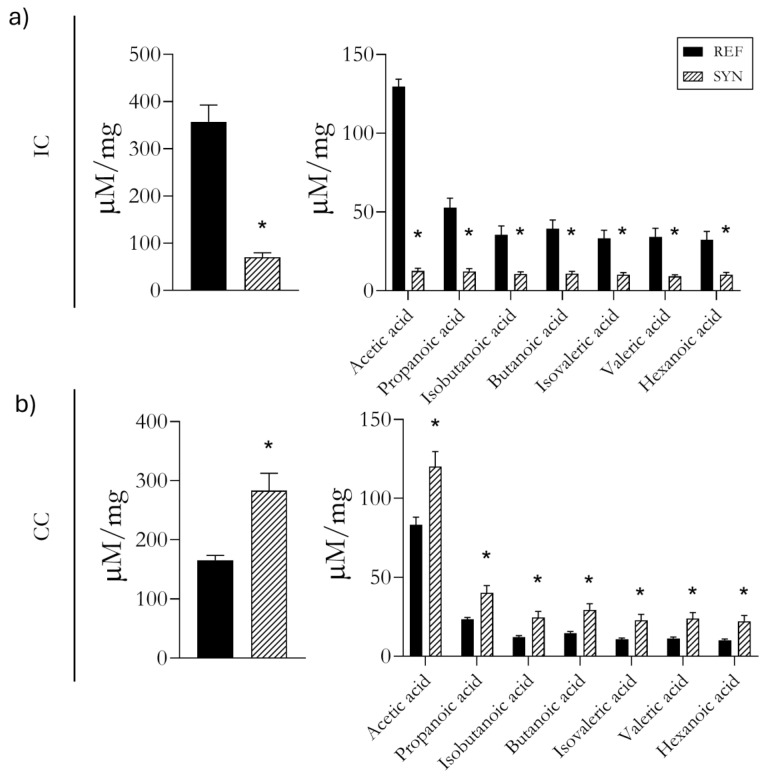
Intestinal and cecal short-chain fatty acid (SCFA) levels at day 28 of life. (**a**) SCFAs in intestine (IC); (**b**) SCFAs in cecum (CC). Results are expressed as mean ± SEM. Statistical differences: * *p* < 0.05 (*n* = 9–11).

The SCFAs at both the intestinal and cecal levels were examined to establish a relationship with the microbiota findings in both compartments (Figure 8). For the IC, a decrease in SCFA production was found, specifically in acetic, propanoic, isobutanoic, butanonic, iso-valeric, valeric, and hexanoic acids (Figure 8a). Conversely, for the CC, the overall SCFA production increased, attributed to higher levels of all the aforementioned acids (Figure 8b). 

In addition, the levels found in the SCFAs were associated with those of the microbiota composition in both the intestine and the cecum, displaying opposite patterns (Supplementary Figure S3). To date, a positive correlation was found for some bacterial groups between the CC and SCFA levels, such as for *Bifidobacterium* and *Faecalibaculum*, whereas *Alistipes* and the *Eubacterium fissicatena* group had a negative correlation between the two. 

### 3.10. Lymphocyte Subset Characterization

The impact of the synbiotic supplementation was also assessed on lymphocytic populations both at the systemic level, in the spleen, and at the mucosal level, in the MLNs. In addition to the main lymphocyte populations, markers related to intestinal migration (αE and CD62L) were also measured, as well as the levels of the cellular activation marker CD25. Furthermore, the expression of the CD8 receptor on T lymphocytes was also assessed (Table 3).

In the spleen, the percentage of B lymphocytes was higher after the SYN supplementation, whereas activated B cell (CD25+) proportion was not altered. The analysis of the T cell subsets showed no changes in the total T cells, including in the TCRαβ+ and the TCRgδ+ subpopulations. However, a reduction in the total T CD4+ and the activated T CD4+ cells was observed after the nutritional intervention. The SYN supplementation reduced the surface cell expression migratory markers in the spleen after the supplementation. An evaluation showed that the lymphocyte populations at the mucosal level did not significantly affect the B or T cell populations. 

### 3.11. Systemic and Mucosal Immunoglobulin Profiles

**Figure 9:**
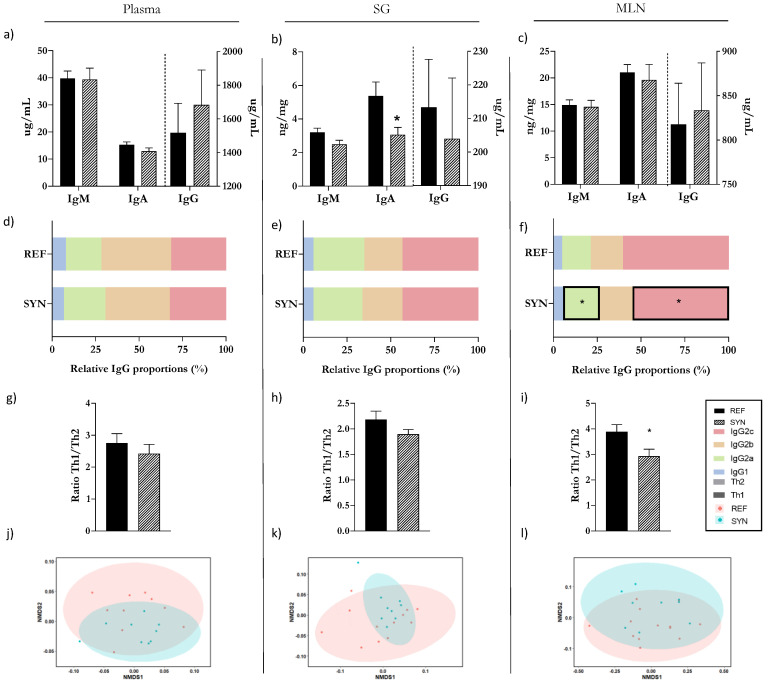
Immunoglobulin profile assessment in plasma, SG, and MLNs at day 28 of life. Total levels of IgM, IgA, and IgG in plasma (**a**), SG (**b**), MLNs (**c**); Relative proportion of IgG subtypes in (**d**) plasma, (**e**) SG, and (**f**) MLNs; Analysis of the Th1/Th2 ratio in plasma (**g**), SG (**h**), and MLNs (**i**). Analysis of non-parametric multidimensional scaling (NMDS) for the Ig profiles based on the BrayCurtis distance in plasma (**j**), SG (**k**), and MLNs (**l**). Results (**a**–**i**) are expressed as mean ± SEM. In NMDS plots (**j**–**l**), each point represents an animal according to ANOSIM testing * *p* < 0.05 vs. REF (*n* = 9–11). SG, salivary gland; MLNs, mesenteric lymph nodes; Ig, immunoglobulin.

The impact of the synbiotic supplementation on the rats’ immunological profiles at both the systemic and mucosal levels was evaluated at day 28 of their life (Figure 9). In all compartments, the most abundant Ig is IgG, followed by IgM, with IgA being the least abundant. The synbiotic nutritional intervention did not result in any significant modifications in the total amount of IgG, IgM, or IgA in their plasma and MLNs. However, the SYN group exhibited a reduction in IgA levels in the SG (Figure 9a–c). In rats, there are four subtypes of IgG (Ig1b, Ig2a, Ig2b, and Ig2c), and the relative proportions of each one were analyzed for each compartment (Figure 9d–f). In plasma, IgG2b is the major Ig, while in the mucosal compartments (the SG and MLNs), the predominant Ig is IgG2c. In the MLNs of the SYN group, a reduction in the relative proportion of IgG2c and an increase in IgG2a were observed. The Th1/Th2 ratio was evaluated by measuring the levels of IgG2b and IgG2c (representing Th1) and IgG1 and IgG2a (representing Th2) (Figure 9g–i). The nutritional intervention resulted in a reduction in the Th1/Th2 ratio only in the MLNs after one week of administration. To provide a comprehensive analysis of the Ig profiles, NMDS graphs were plotted (Figure 9j–l). After the synbiotic supplementation, there was no significantly different distribution of the clusters in any of the evaluated compartments.

## 4. Discussion

The present study confirms that synbiotics in early life contribute to the healthy development of the immune system, mainly affecting intestinal maturation and microbiota colonization. 

Early-life development is highly influenced by environmental factors. During the first 6 months of life, the World Health Organization (WHO) recommends exclusively breastfeeding for proper neonate maturation [39]. BM composition is rich in bioactive compounds that contribute to the nourishment of the infant while conferring passive immunity. After weaning, the immune system continues the maturation process, and nutritional habits contribute to the infant’s development [40]. In recent years, the introduction of pre-, pro-, and synbiotics in infants’ diets has been further studied as an effective strategy to reinforce their immune systems [41].

The gastrointestinal tract (GIT) is one of the principal channels of infections due to its constant exposure to external antigens. The reduction in the incidence of intestinal infections after boosting the functionality of the GIT with pro-, pre-, and synbiotics has gained interest over the last decade. Special focus has been given to the interaction between biotics supplementation and the intestinal microbiota. The modulation of microbiota functionality through biotics contributes to the maintenance of a healthy status and reduces the development of asthma [42], necrotizing enterocolitis (NEC) [43], eczema [44], and inflammatory bowel diseases [45]. *Bifidobacterium* species have been widely studied since they modulate intestinal microbiota. *B. breve* M-16V is a probiotic strain isolated from the infant gut that prevents NEC and reduces the incidence of infectious and atopic diseases when administered in early life [46,47]. Thus, *B. breve* M-16V metabolizes the human milk oligosaccharides (HMOs), which act as prebiotic substrates for the neonatal microbiota of the GIT [48]. 

Apart from the HMOs, *Bifidobacterium* species also metabolize non-milk oligosaccharides, such as GOS, FOS, inulin, lactulose, or their combination [49]. Among the most studied probiotic substances that are metabolized by *B. breve* M-16V are scGOS/lcFOS, which contribute to the growth of the probiotic strain [50,51]. Due to the attributed benefits of the synbiotic mixture of *B. breve* and scGOS/lcFOS, their combination is used for infant formulas to mimic BM [26,52]. Considering the above, we hypothesized that supplementation with *B. breve* M-16V and scGOS/lcFOS given to dams during the gestation and lactation periods and given to pups after the weaning period may improve the maturation of the rats’ immune system. This period in rats is equivalent to the first 1000 days in humans, which has been defined as relevant for infant development [20].

In the present study, the animals’ body weight and food intake were monitored daily. From birth, similar growth patterns were observed in both the REF and SYN groups, suggesting that the maternal and infant supplementation with the synbiotic did not affect the rats’ overall growth. Food intake as well as organ weight were similar in both groups. Other studies based on the administration of synbiotics have shown that infants’ growth patterns are not affected [53,54,55,56]. The lack of impact on the infant’s body weight after the maternal intervention and the continuation of the synbiotic into the infant’s early childhood seems to show the safety of this nutritional intervention [55,57,58].

Regarding adiposity, the supplementation with the synbiotic mixture did not affect the weight of the rats’ WAT but increased the weight of their BAT. However, a histological analysis of both tissues revealed no significant morphological changes. In metabolic pathologies such as obesity or diabetes, the administration of synbiotics has improved previously impaired lipid parameters through the modulation of gut microbiota [59,60]. However, fewer studies have used synbiotics under healthy conditions. The study by Hosseinifard et al. evaluated the impact of a synbiotic (composed of a *Lactobacillus* strain and inulin) in healthy rats and concluded that the synbiotic contributed to the amelioration of and reduction in oxidative parameters [58]. Further studies are necessary to evaluate the impact of the combination of B. *breve* M-16V and scGOS/lcFOS on the molecular pathways of BAT metabolism.

The observed trophic effect on the SI due to the synbiotic supplementation led to the analysis of its microscopic structures and the maturation stage of this organ. The HE staining showed an increase in the width and area of the villi, an increase in the crypt depth, and a higher number of goblet cells per villi. Additionally, the gene expression of two inverse maturation markers, *FcRn* and *Blimp1*, was reduced after the supplementation. In the poultry industry, supplementation with synbiotics has been widely introduced to increase production and animal performance [61]. One of the most desired effects when supplementing animal diets is the reduction in gastrointestinal infections by boosting intestinal microstructures [61]. In addition, an increase in the villi height suggests a more effective absorption of nutrients due to the higher surface area [62]. In the crypts, the stem cells are involved in the renewal of the epithelial cells, and deeper crypts are associated with increased tissue turnover, which is essential for maintaining the health and functionality of the SI, as a quicker turnover helps it to replace the damaged cells and to respond better to inflammation [63]. Moreover, *Blimp1* is expressed during fetal development and is involved in promoting adaptive and innate immune cell differentiation [64]. After birth, it is downregulated until reaching a low adult profile [65]. Additionally, it has recently been recognized that *FcRn*, responsible for transporting IgG across the placenta during fetal development and milk IgG during lactation, provides humoral immunity to newborns [66]. After weaning, *FcRn* expression is reduced, which is an indicator that there is no need for more milk IgG absorption. So, despite the crucial roles that these markers play in early life, their expression diminishes after weaning, when maturation is achieved [65,66,67]. Consequently, the observed morphological changes and the reduction in these inverse maturation markers following supplementation indicates that synbiotics enhance intestinal maturation.

Additionally, genes associated with intestinal barrier functionality were also studied. The synbiotic administration induced an increase in the *Zo1* gene and in the *Tlr9* gene after one week of supplementation. These molecules are implicated in intestinal immunity, and their modulation through diet has been proven to reduce pathogenic challenges [65]. The TJ proteins contribute to the maintenance of the physiological barrier, modulating the transport of nutrients, water, and ions through the intestinal epithelium [68]. Probiotic and prebiotic supplementation has been linked to the modulation of the expression of TJ proteins by preventing its disruption under pathogenic conditions [69,70]. Additionally, weaning is a stressful situation for animals, during which the intestinal barrier suffers an imbalance. In this regard, supplementing weaned pigs with probiotics enhanced the intestinal barrier function, increasing the expression of *Zo1* and favoring epithelial barrier integrity [71]. TLRs are involved in the recognition of microbial components, activating immunological responses [72]. The effect of *B. breve* in the modulation of the TLR signaling pathway by increasing *Tlr9* and inducing an anti-inflammatory status has been widely described [73,74]. Taken together, our results suggest that the synbiotic mixture used maintains intestinal barrier functionality while stimulating TLR immunological responses. 

In the intestinal compartment, the IgA interacts with the pathogenic bacteria, neutralizes them, and prevents infections. The effect of probiotics, prebiotics, and synbiotics on intestinal sIgA under pathogenic and healthy conditions has been studied [75,76]. However, under healthy conditions, it has been less analyzed [77]. Our results suggest that the present synbiotic supplementation reduced the IgA mRNA levels even though this result was not reflected in terms of protein levels. This phenomenon suggests that different transcriptional modifications occur, but that they do not modify the protein levels in the intestine [78].

In the cecum, the IgA binds to the cecal bacteria, neutralizes them, and facilitates their elimination [79]. Our synbiotic supplementation induced a reduction in the cecal free sIgA, but an increase in bacteria bound to IgA is seen. This result suggests that free sIgA was reduced due to the IgA binding to bacteria after the synbiotic supplementation.

Although there was a positive impact on the Ig profile in the gastrointestinal tract, a lower impact was observed on the overall Ig profile. Only a punctual reduction in the sIgA in the SG and IgG2c in the MLNs was observed, along with an increase in IgG2a in the MLNs. Despite these changes, the maturation ratio measured by the Th1/Th2 ratio responses was not affected in the plasma or in the SG. However, in the intestinal mucosal compartment in the MLNs, the Th1/Th2 ratio was reduced, indicating that the synbiotic supplementation did not shift toward a Th1 response. At birth, the immune system is biased toward a Th2 response; as it matures, the immune response switches towards a Th1 response [80]. However, this is an interesting fact, as it has to be considered that during weaning, a great number of new antigens arrive in the intestine and a pro-inflammatory state is induced there. The SYN group seems to control this pro-inflammatory state while allowing for the same Th1/Th2 systemic balance, indicating the same level of maturity as that of the REF animals but with a lower intestinal state of alert. These mucosal results seem to be more in line with the positive impact of the synbiotic on the maturation markers evaluated at the intestinal gene expression level. 

The supplementation did not affect the overall lymphocyte subsets in the MLNs or in the spleen. Different studies have demonstrated an impact on lymphocyte markers after synbiotic supplementation [81,82]. However, it needs to be considered that the effects of these synbiotics are dose- and strain-specific. Moreover, the duration of these nutritional studies was longer than a week and did not evaluate supplementation administered to the mother. Further studies considering long-term supplementation could elucidate the same results after nutritional interventions. 

Regarding the main changes in the microbiota composition in the intestine, an increase in *Bifidobacterium* and *Turicibacter* and a reduction in *Ligilactobacillus* were observed after the synbiotic supplementation. It is widely known that breastfeeding contributes to a higher level of *Bifidobacterium* colonization in the infant’s gut compared to that in a formula-fed infant. However, in this case, the higher levels of *Bifidobacterium* may be derived, at least partly, from the pups taking supplements directly after weaning. Yin et al. supplemented weaned pig with oligosaccharides and found an increase in *Turicibacter*, which may mediate host metabolism and physiological functions [71,83]. Although the data on intestinal microbiota changes after synbiotic supplementation are limited, our results confirm that synbiotic supplementation modulates the intestinal microbiota, favoring its colonization and improving its functionality.

In the cecum, the abundance of *Bifidobacteriaceae* was increased, and a reduction in *Staphylococaceae* was found after the synbiotic intervention. This result is in line with other studies showing that some synbiotics contribute to reducing the proportion of *Staphylococaceae* [84,85]. The genera of *Bifidobacterium*, *Blautia*, *Faecalibaculum*, and *Lactobacillus* were increased after the supplementation. The cessation of breastfeeding is linked with the increase in these genera [86]. *Bifidobacterium* and *Lactobacillus* are well known as probiotics for their potential to produce SCFAs and bacteriocins. However, the importance of *Blautia* has been of particular interest lately due to its ability to alleviate inflammatory and metabolic diseases and for its antibacterial activity [87,88]. The increase in the *Bifidobacterium*, *Blautia*, *Faecalibaculum*, and *Lactobacillus* genera was reported previously by Qi Zhang et al., whose study involved a synbiotic (*Bifidobacterium* and FOS) added to an in vitro fermentation model [89]. All these data, joined with the presence of the administered probiotic strain for the CC, corroborate the fact that supplementation contributes to the colonization of cecal microbiota after one week. 

The functionality of microbiota is highly linked to the fermentation of unfermented fibers, including the fermentation of the GOS/FOS. The fermentation of undigestible carbohydrates and the release of SCFAs occur throughout the entire GIT [90]. However, the cecum has been described as the most active place for the production of SCFAs [91,92]. According to our results, the reduction in the SCFAs in the intestine could be associated with the lower level of bacterial diversity compared to that in the cecum and its rapid absorption by intestinal cells. However, a more in-depth investigation into the mechanism of SCFAs’ production and their functionality in the intestine is needed to complete our results.

In the cecum, the overall increase in SFCA production after the supplementation may be attributed to an increase in the most abundant acids—acetic and propionic—which are produced in large quantities by *Bifidobacterium* populations [50,93]. Also, the SCFAs’ increase could be linked to *Faecalibaculum*, which has been recently described as an SCFA producer [94]. Additionally, other SCFAs, such as valeric, isovaleric, isobutyric, and hexanoic, are linked to *Clostridium difficile* strains [95]. However, we did not find any differences in the proportions of *Clostridium* genera after the supplementation. 

Apart from the positive results of the synbiotic intervention, some limitations were found within this study. In general, preclinical studies are really useful to understand the developmental changes required in early life. Additionally, animal models contribute to clarify the biological mechanisms and interactions of the infant with external agents in their maturation process. However, the integration of pre-clinical and clinical data may be difficult; for example, microbiota colonization differs among species. In the present work, the potential contribution of synbiotic exposure during early life to the healthy maturation of infants was evaluated. Once the principal benefits of synbiotic exposure for infant maturation are established, a further step could be to get closer to the human perspective by including the principal infections or pathological conditions that occur in infancy. Consequently, the potential effects of synbiotic exposure during early life could be analyzed under conditions more similar to those of humans. Additionally, it is necessary to clarify which period—gestation, lactation, or early childhood—is the most critical for offspring development. This would help us determine the optimal period for synbiotic nutritional interventions to maximize supplementation benefits.

## 5. Conclusions

This study provides valuable insights into the potential of *B. breve* M-16V and scGOS/lcFOS intervention during gestation, lactation, and early life. It positively shapes the maturation and functionality of the intestinal barrier and microbiota, ultimately contributing to enhanced gastrointestinal immunity in offspring. The influence of the maternal diet during pregnancy and lactation also contributes to the maturation of the infant, which is highly important for the first 1000 days of life. Future investigations are needed to explore the long-term consequences of maternal synbiotic supplementation for offspring health from infancy into childhood.

## Figures and Tables

**Table 1:** Animal growth parameters in 28-day-old animals.

**Body**	**REF**	**SYN**
Body length (cm)	13.58 ± 0.11	13.26 ± 0.15
Body/tail length ratio	1.42 ± 0.02	1.55 ± 0.14
BMI (g/cm^2^)	0.36 ± 0.01	0.35 ± 0.05
Lee index (g^0.33^/cm × 10^3^)	298.82 ± 1.80	295.93 ± 3.15
**Organs**	**REF**	**SYN**
Spleen (%)	0.36 ± 0.01	0.36 ± 0.01
Thymus (%)	0.44 ± 0.02	0.44 ± 0.02
Kidney (%)	0.62 ± 0.01	0.59 ± 0.01
Heart (%)	0.58 ± 0.01	0.60 ± 0.03
Liver (%)	4.49 ± 0.04	4.09 ± 0.46
Salivary gland (%)	0.24 ± 0.09	0.16 ± 0.01
Stomach (%)	0.71 ± 0.02	0.73 ± 0.02
Cecum (%)	0.33 ± 0.02	0.30 ± 0.01
Small intestine (%)	4.18 ± 0.07	4.61 ± 0.19 *
Intestine length (cm)	67.04 ± 2.14	72.39 ± 2.95 #
Intestine width (cm)	0.85 ± 0.07	1.15 ± 0.05 *
Area (cm^2^)	56.26 ± 5.03	83.90 ± 6.59 *

Data are expressed as mean ± SEM. Statistical differences: * *p* < 0.05 vs. REF, # *p* < 0.1 vs. REF (*n* = 9–11).

**Table 2:** Hematological variables at day 28 of life.

	REF	SYN
Leukocytes (×10^9^/L)	4.84 ± 0.93	2.78 ± 0.2 *
Lymphocytes (×10^9^/L)	3.09 ± 0.42	1.89 ± 0.18 *
Monocytes (×10^9^/L)	0.27 ± 0.12	0.11 ± 0.01
Granulocytes (×10^9^/L)	1.47 ± 0.40	0.78 ± 0.08 *
Erythrocytes (×10^12^/L)	5.41 ± 0.25	5.38 ± 0.12
HGB (g/L)	101.79 ± 4.02	101.22 ± 2.47
HCT (%)	29.76 ± 1.47	29.79 ± 0.93
MCV (fL)	55.01 ± 0.62	55.34 ± 0.69
MCH (pg)	18.99 ± 0.40	18.74 ± 0.11
Platelets (×10^9^/L)	234.54 ± 28.88	182.89 ± 41.62

Data are expressed as mean ± SEM. Statistical differences: * *p* < 0.05 vs. REF (*n* = 9–11). HGB, hemoglobin; HCT, hematocrit; MCV, mean cell volume; MCH, mean cell hemoglobin.

**Table 3:** Lymphocyte subsets’ phenotype in the spleen and MLNs at day 28 of life.

	Spleen		MLNs	
	REF	SYN	REF	SYN
B cells (CD45RA+)	25.44 ± 2.26	35.14 ± 0.64 *	17.05 ± 1.58	17.85 ± 1.17
% CD25+	2.51 ± 0.43	1.40 ± 0.26	2.86 ± 0.19	2.10 ± 0.32
% CD62L+	46.15 ± 2.69	39.52 ± 2.78	65.84 ± 5.24	70.23 ± 2.32
% αE+	2.95 ± 0.58	4.04 ± 0.84	0.09 ± 0.03	0.06 ± 0.01
T cells (TCRαβ+NK- and TCRgδ)	54.01 ± 6.12	36.94 ± 1.21	79.17 ± 1.86	78.77 ± 1.04
TCRαβ+ NK-	50.60 ± 6.33	33.20 ± 1.06	75.03 ± 1.49	75.86 ± 1.07
% CD8	24.24 ± 0.75	24.51 ± 0.49	27.24 ± 1.20	28.78 ± 0.69
TCRgδ+	3.40 ± 0.31	3.74 ± 0.31	4.14 ± 1.08	2.90 ± 0.15
% CD8	55.50 ± 4.29	59.96 ± 3.62	57.57 ± 1.65	54.54 ± 3.07
CD4+ CD8-	43.18 ± 3.62	29.96 ± 0.91 *	58.81 ± 1.40	57.84 ± 1.02
% CD25+	7.68 ± 0.40	5.33 ± 0.44 *	7.67 ± 0.32	8.56 ± 0.39
% CD62L+	80.69 ± 0.91	77.31 ± 1.53	61.70 ± 4.59	49.18 ± 9.02
% αE+	1.30 ± 0.15	1.49 ± 0.26	3.51 ± 1.65	11.46 ± 3.55
CD8+ CD4-	17.59 ± 0.52	16.20 ± 0.55	19.82 ± 0.82	20.12 ± 0.53
% CD25+	4.29 ± 0.31	3.56 ± 0.47	3.77 ± 0.38	4.03 ± 0.40
% CD62L+	66.59 ± 2.62	65.13 ± 1.65	68.40 ± 1.55	67.75 ± 3.99
% αE+	0.89 ± 0.11	0.85 ± 0.14	1.27 ± 0.21	0.99 ± 0.25
CD4+ CD8+	1.55 ± 0.67	1.42 ± 0.13	1.61 ± 0.06	1.59 ± 0.09
% CD25+	73.51 ± 6.20	90.4 ± 3.63	53.86 ± 5.27	45.68 ± 3.79
NK (TCRαβ- NK+)	5.31 ± 1.36	6.42 ± 0.71	1.09 ± 0.11	1.10 ± 0.07
% CD8	7.82 ± 1.78	6.28 ± 0.75	10.95 ± 1.19	13.50 ± 0.06
NKT (TCRαβ+ NK+)	2.59 ± 0.40	3.11 ± 0.30	1.87 ± 0.11	1.63 ± 0.10
% CD8	45.83 ± 1.81	48.71 ± 2.04	61.59 ± 3.29	70.12 ± 1.02
αE+	1.17 ± 0.11	1.65 ± 0.20	0.66 ± 0.10	0.60 ± 0.14
CD62L+	62.72 ± 3.08	54.44 ± 1.24 *	72.16 ± 1.82	70.73 ± 2.07

Data are expressed as mean ± SEM. Statistical differences: * *p* < 0.05 vs. REF (*n* = 9–11).

## Data Availability

The original contributions presented in the study are included in the article and Supplementary Materials, further inquiries can be directed to the corresponding author.

## Supplementary Materials

The following supporting information can be downloaded at: https://www.mdpi.com/article/10.3390/foods13132058/s1, Figure S1: Experimental design diagram of the nutritional intervention; Figure S2: Analysis of non-parametric multidimensional scaling (NMDS) for the microbiota profiles; Figure S3: Correlation between the principal common genera in the intestinal content (IC) and the cecal content (CC) and the levels of short chain fatty acids (SCFAs) of both compartments; Table S1: Description of the specific TaqMan primers AB; Table S2: Lower limits of detection of Procartaplex; Table S3: DESQ2 test in cecal content (CC) and intestinal content (IC).
