# Supplementary material for: Early-Life Supplementation Enhances Gastrointestinal Immunity and Microbiota in Young Rats
Source: Foods. 2024 Jun 28;13(13):2058. doi: 10.3390/foods13132058 (PMC11241808; doi:10.3390/foods13132058)
Supplement: Supplementary file 1 [file foods-13-02058-s001.zip › foods-3044759-supplementary.pdf]

Supplementary Figures

Figure S1

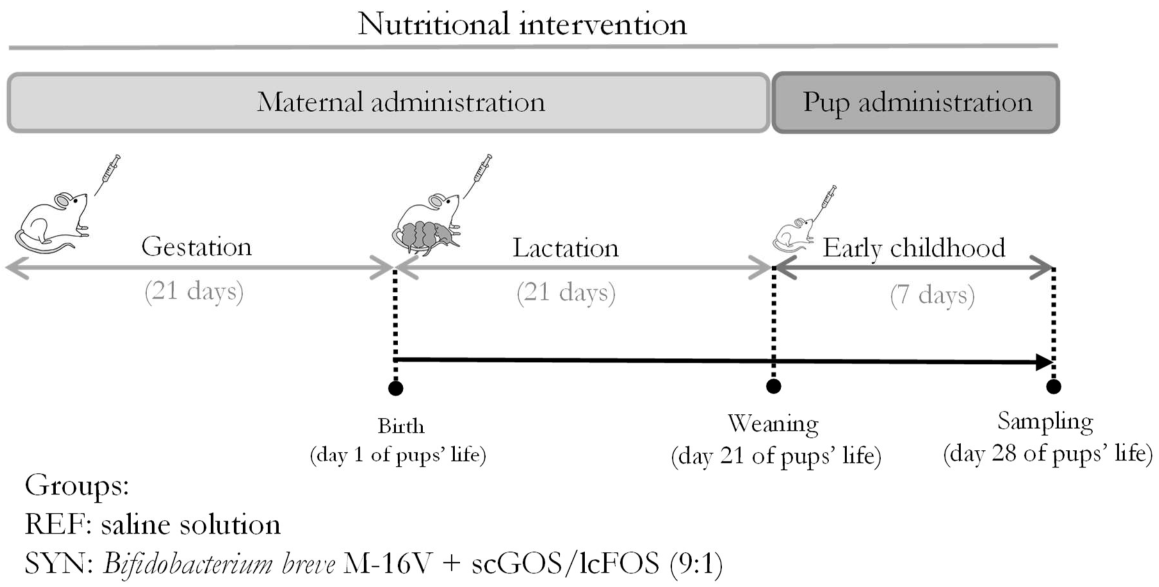

Figure S1. Experimental design of the study. Animals were divided into two groups reference (REF) which received the matching volume of saline solution and synbiotic (SYN). The number of pups for each group was 9 and 11, respectively.

Figure S2.

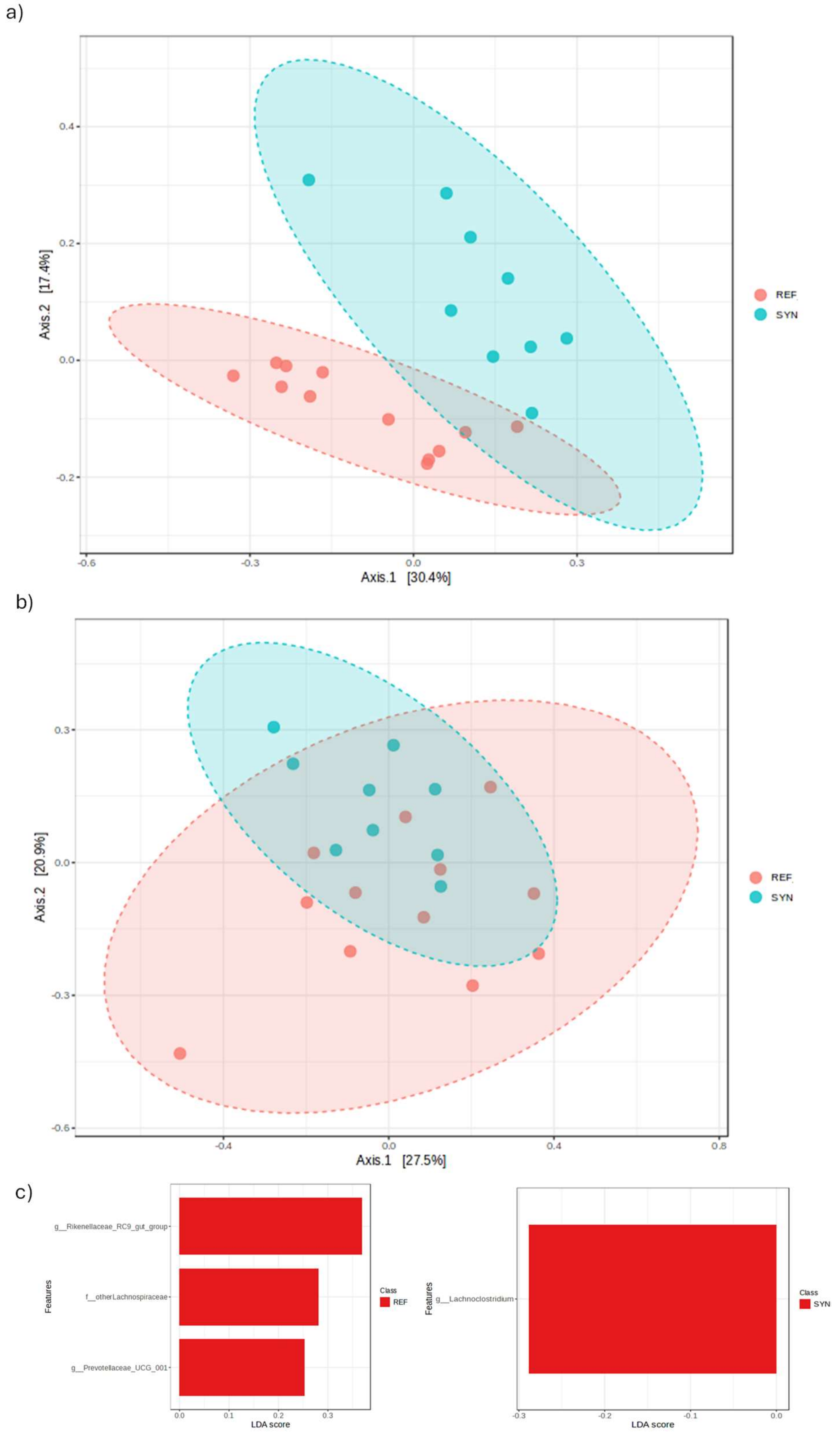

Figure S2. Analysis of non-parametric multidimensional scaling (NMDS) for the microbiota profiles based on the Bray-Curtis distance in (a) IC and (b) CC. (c) Taxonomic biomarkers in both dietary groups using LEfSe plot. Each point in NMDS represents an animal by ANOSIM test. Statistical differences: *$p < 0.05$ vs REF (n=11-16).

Figure S3.

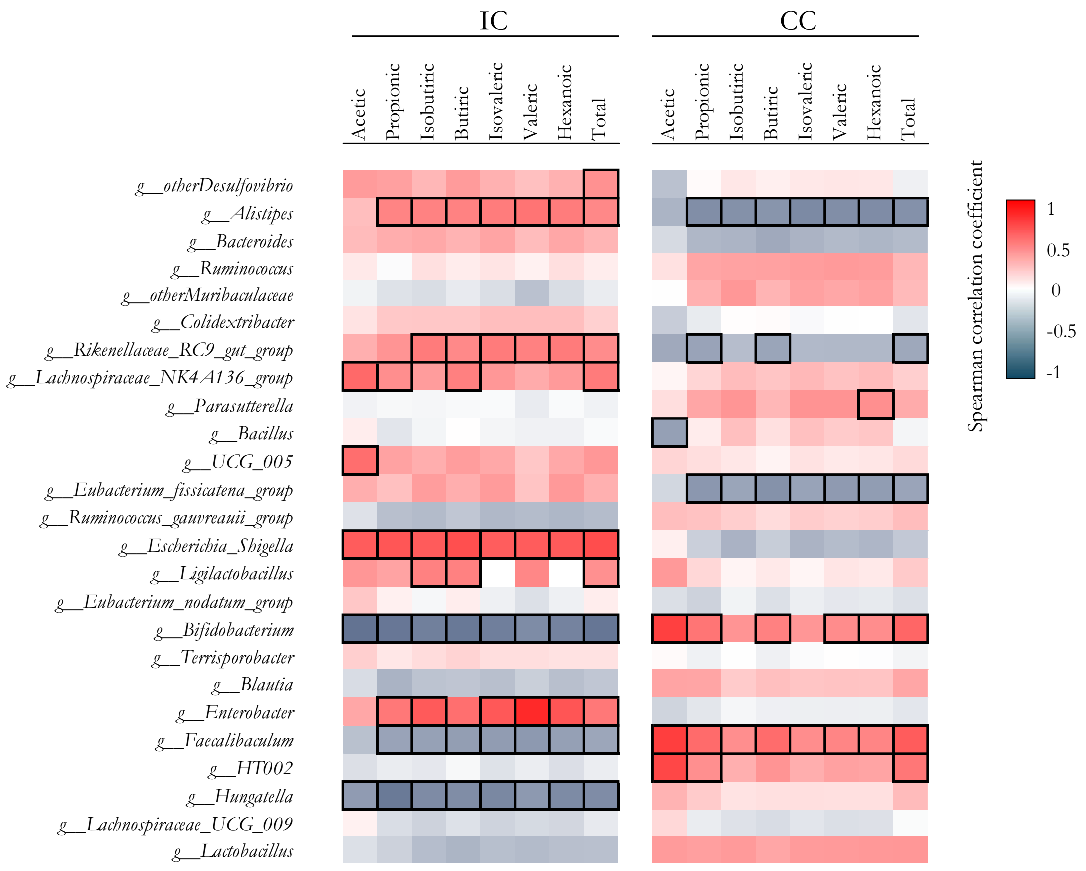

Figure S3. Correlation between the shared genera of the intestinal content (IC) and cecal content (CC) with the levels of the short chain fatty acids (SCFAs) of each compartment. The Spearman correlation coefficient is represented in the heat map following the color in the legends. Correlations with statistical significance ($p < 0.05$) are shown in a bold frame (n=9-11).

Supplementary Tables.

Supplementary Table S1. Description of the specific TaqMan primers AB

Gene	Reference
Tlr2	Rn02133647_s1, I
Tlr3	Rn01488472_g1, I
Tlr4	Rn00569848_m1, I
Tlr5	Rn04219239_s1, I
Tlr7	Rn01771083_s1, I
Tlr9	Rn01640054_m1, I
Muc2	Rn01498206_m1, I
Muc3	Rn01481134_m1, I
Ocln	Rn00580064_m1, I
Cldn2	Rn02063575_s1, I
Cldn4	Rn01196224_s1, I
ZO1	Rn02116071_s1, I
IgA	331943, made to order
Blimp1	Rn03416161_m1, I
Fcgrt	Rn00566655_m1, I, encoding for FcRn
Gusb	Rn00566655_m1, I

I, inventoried

Supplementary Table S2. Lower limits of Ig detection of Procartaplex.

Immunoglobulin	Lower limits of detection
IgA	0.58 ng/mL
IgM	0.2 ng /mL
IgG1	1.70 ng/mL
IgG2a	1.73 ng/mL
IgG2b	2.67 ng/mL
IgG2c	3.67 ng/mL

Supplementary Table S3. Table DESQ2 of cecal content (CC) and intestinal content (IC).

		log2FC	lfcSE	Pvalues,	FDR
**CC**	***g__Eisenbergiella***	8.6576	1.0195	2.036E-17	1.527E-15
	g__Candidatus_Stoquefichus	-9.4724	1.8487	2.996E-7	1.1235E-5
	g__HT002	4.0062	0.92113	1.3661E-5	3.4152E-4
	g__Faecalibaculum	5.2232	1.3412	9.8382E-5	0.0018447
	g__NK4A214_group	-3.7307	0.98073	1.4236E-4	0.0021355
	g__Bifidobacterium	4.2101	1.1336	2.0397E-4	0.0025496
	g__Rikenellaceae_RC9_gut_group	-1.7872	0.48891	2.5672E-4	0.0027506
	g__UCG_009	-3.335	0.94976	4.4577E-4	0.0041791
	g__Marvinbryantia	4.0049	1.2165	9.9382E-4	0.0077699
	f__otherLachnospiraceae	-1.4485	0.44153	0.001036	0.0077699
	g__Ruminococcus_gauvreauii_group	1.9864	0.6609	0.00265	0.018068
	g__Family_XIII_AD3011_group	-2.4779	0.9138	0.0066948	0.041843
	Not_Assigned	-1.085	0.44193	0.014079	0.081225
	g__Blautia	2.6647	1.1007	0.015482	0.082941
	g__Tuzzerella	-3.7756	1.7751	0.033421	0.15419
	g__Ligilactobacillus	1.2115	0.57356	0.034666	0.15419
	g__Eubacterium_xylanophilum_group	-3.7618	1.7963	0.036243	0.15419
	g__Intestinimonas	-0.96904	0.46461	0.037006	0.15419
	g__Lactobacillus	4.3247	2.1596	0.045227	0.17853
IC	***g__Escherichia_Shigella***	-3.237	0.74934	1.562E-5	5.7792E-4
	g__Turicibacter	2.4926	0.68571	2.7793E-4	0.0051416
	g__Hungatella	5.4855	1.607	6.414E-4	0.0079106
	g__Lactococcus	-1.9655	0.63821	0.0020723	0.019169
	g__Enterobacter	-3.3948	1.1707	0.0037344	0.027635
	g__Lachnoclostridium	2.5312	1.0294	0.013935	0.085934
	g__Ligilactobacillus	-1.4179	0.60834	0.019768	0.10449
	g__Lachnospiraceae_NK4A136_group	-2.4662	1.2206	0.043325	0.1924
	g__Erysipelatoclostridium	2.342	1.178	0.046799	0.1924
	g__otherDesulfovibrio	-3.3991	1.8096	0.060331	0.22323
